# PEAK 2.0: OPERATIONALIZING PERSON-CENTERED CARE AIDS NURSING HOMES IMPLEMENT AND SUSTAIN PRACTICES

**DOI:** 10.1093/geroni/igz038.2050

**Published:** 2019-11-08

**Authors:** Laci Cornelison, Gayle Doll, Maggie Syme, Migette Kaup

**Affiliations:** Kansas State University, Manhattan, Kansas, United States

## Abstract

Pay-for-performance programs to incentivize quality are on the rise nationally (Werner, Konetzka, & Polsky, 2013 & Arling, Job, & Cooke, 2009). Kansas initiated a Medicaid P4P program to incentivize person-centered care (PCC) beginning in 2012 called PEAK 2.0. This program created an operationalized definition of PCC through stakeholder collaboration and research outcomes (Harris, Poulsen, & Vlangas, 2006). Homes enrolled in the program undergo both self-evaluation and objective external evaluation based on the operationalized definition. These key features inherent in the PEAK 2.0 program make up has aided homes to implement PCC as well as, the ability to research homes that have implemented PCC in a new a different way than ever before.

